# Irbesartan in Marfan syndrome (AIMS): a double-blind, placebo-controlled randomised trial

**DOI:** 10.1016/S0140-6736(19)32518-8

**Published:** 2019-12-21

**Authors:** Michael Mullen, Xu Yu Jin, Anne Child, A Graham Stuart, Matthew Dodd, José Antonio Aragon-Martin, David Gaze, Anatoli Kiotsekoglou, Li Yuan, Jiangting Hu, Claire Foley, Laura Van Dyck, Rosemary Knight, Tim Clayton, Lorna Swan, John D R Thomson, Guliz Erdem, David Crossman, Marcus Flather, AIMS Investigators, John Dean, Bartosz Was, Heather Gow, Jane Murray, Mariella D'Allessandro, Michael Christie, Patricia Cooper, Philip Booth, Sharon Burns, Yvonne Paterson, Ashish Chikermane, Anthony Assing, Catherine Cotter, Gillian Atkins, Helen Williamson, Justin Barclay, Alan Jennison, Alex Henderson, Anna McSkeane, Helen Fairlamb, Julie Kelly, Nicola Kelsall, Scott Prentice, John O'Sullivan, Alison Head-Baister, Angela Phillipson, Anna Johnson, D Crossland, Jack Oliver, Jade Davison, Jill Wake, Louise Quinn, Maureen Foreman, Vera Wealleans, Niki Walker, Alexis Duncan, Evelyn Tibbs, Ruth Kelly, Sachin Khambadkone, Bridget Zotti, Cassie Brady, Elena Cervi, Ella Field, Eszter Szepezvary, Florence Mantey, Gillian Riley, Heather Titmus, Ilaria Bo, Juan Pablo Kaski, Loren Green, Nigel Jones, Rebecca Banks, Christopher Kiesewetter, Sujeev Mathur, Alessandra Frigiola, Alex Savis, Holly Belfield, Josephine Guzman, Julia Harris, Karen Wilson, Kelly Peacock, Kirsty Gibson, Paul Wellman, John Simpson, Saleha Kabir, Sitali Mushemi, Michael Stewart, Bev Atkinson, Cath Richardson, Elaine Leng, Paul Brennan, Annabel Nixon, Collette Spencer, James Oliver, Jan Forster, Louise Turner, Samantha Bainbridge, Anna Maria Choy, Adelle Dawson, Gwen Kiddie, Heather Kerr, Ify Mordi, Jackie Duff, Jacqueline Dunlop, Jonathan Berg, Pauline Armory, Leisa Freeman, Amir Anwar, Charles Graham, Clare London, Gail Healey, Ian Gallagher, Mary Ilsley, Rizwan Ahmed, Sheila Wood, Nigel Wheeldon, Cecilia Mason, Farook Nassim, Janet Middle, Justin Adams, Karen Angelini, Kay Housley, Kim Ryalls, Michael Agyemang, Rachel Walker, Robina Batigan, Tina Bennett, Paul Clift, Amor Mia Alvior, Annette Nilsson, Carole Green, Charlotte Crook, Connie Becani Palmer, Elizabeth Dwenger, Phillipa Doherty, Rebecca Igbokwe, Saba Sharif, Sonia MacDonald, Cathy West, Kevin Kirby, Nitha Naqvi, Sophie Welch, Suad Warsama, Wei Li, Zohreh Farzad, Ben Smith, Victoria Murday, Alexis Duncan, Eamonn Murtagh, Emma Adams, Lesley Armour, Stuart Lilley, Bejal Pandya, Amy Richards, Mervyn Andiapen, Rebecca Macrae, Maite Tome, Carmel Hutchinson, Kameka Angulo, Rooba Kauppayamootoo, Sabiha Gati, Elizabeth Cruddas, William G Newman, Catherine Breen, Dhavendra Kumar, Dirk G Wilson, Adele Farrugia, Alan Fraser, Jayne Sumers, Jessie Powell, Julie Edwards, Terese Hale, Zoe Boult, Aisling Carroll, Gruschen Veldtman, Andrew Ho, David Black, Lisa Fletcher, Sue Mapstone, Tara Bharucha, Gary Marsh, Joanne Jones, Karen Sheehan, Kathleen Selway, Kirsty Stevenson, Martin Nelson, Rebecca Fairweather, Stephanie Curtis, Sue Simpson, Martin Denvir, Audrey White, Jill Steven, Joanna Munro, Wayne Lam, William Toff, Mario Petrou, Paul Silcocks, Raymond MacAllister

**Affiliations:** a Barts Heart Centre, Barts Health NHS Trust, London, UK; b Department of Cardiovascular Medicine and Devices, Queen Mary University, London, UK; c Core Echo Lab, Nuffield Division of Clinical Laboratory Sciences, Radcliffe Department of Medicine, University of Oxford, Oxford, UK; d Oxford Heart Centre, John Radcliffe Hospital, Oxford University Hospitals NHS Foundation Trust, Oxford, UK; e Molecular and Clinical Sciences Research Institute, St George's University of London, London, UK; f Heart Institute, University of Bristol, Bristol, UK; g Clinical Trials Unit, Department of Medical Statistics, London School of Hygiene & Tropical Medicine, London, UK; h Department of Life Sciences, University of Westminster, London UK; i School of Medical Sciences, Örebro University, Örebro, Sweden; j Ultrasound Department, Wuhan Children's Hospital, Tongji Medical School, Huazhong University of Science and Technology, Hubei, China; k NHS Blood and Transplant, Cambridge, UK; l Department of Adult Congenital Heart Disease, Royal Brompton and Harefield NHS Foundation Trust, London, UK; m Toronto Congenital Cardiac Centre for Adults, Toronto, Canada; n Department of Cardiology, Leeds General Infirmary, Leeds, UK; o Department of Cardiology, Acibadem International Hospital Istanbul, Turkey; p School of Medicine, Acibadem University, Istanbul, Turkey; q School of Medicine, University of St Andrews, St Andrews, UK; r Norwich Medical School, University of East Anglia, Norfolk, Norwich, UK; s Cardiology Department, Norfolk and Norwich University Hospital, Norwich, UK

## Abstract

**Background:**

Irbesartan, a long acting selective angiotensin-1 receptor inhibitor, in Marfan syndrome might reduce aortic dilatation, which is associated with dissection and rupture. We aimed to determine the effects of irbesartan on the rate of aortic dilatation in children and adults with Marfan syndrome.

**Methods:**

We did a placebo-controlled, double-blind randomised trial at 22 centres in the UK. Individuals aged 6–40 years with clinically confirmed Marfan syndrome were eligible for inclusion. Study participants were all given 75 mg open label irbesartan once daily, then randomly assigned to 150 mg of irbesartan (increased to 300 mg as tolerated) or matching placebo. Aortic diameter was measured by echocardiography at baseline and then annually. All images were analysed by a core laboratory blinded to treatment allocation. The primary endpoint was the rate of aortic root dilatation. This trial is registered with ISRCTN, number ISRCTN90011794.

**Findings:**

Between March 14, 2012, and May 1, 2015, 192 participants were recruited and randomly assigned to irbesartan (n=104) or placebo (n=88), and all were followed for up to 5 years. Median age at recruitment was 18 years (IQR 12–28), 99 (52%) were female, mean blood pressure was 110/65 mm Hg (SDs 16 and 12), and 108 (56%) were taking β blockers. Mean baseline aortic root diameter was 34·4 mm in the irbesartan group (SD 5·8) and placebo group (5·5). The mean rate of aortic root dilatation was 0·53 mm per year (95% CI 0·39 to 0·67) in the irbesartan group compared with 0·74 mm per year (0·60 to 0·89) in the placebo group, with a difference in means of −0·22 mm per year (−0·41 to −0·02, p=0·030). The rate of change in aortic Z score was also reduced by irbesartan (difference in means −0·10 per year, 95% CI −0·19 to −0·01, p=0·035). Irbesartan was well tolerated with no observed differences in rates of serious adverse events.

**Interpretation:**

Irbesartan is associated with a reduction in the rate of aortic dilatation in children and young adults with Marfan syndrome and could reduce the incidence of aortic complications.

**Funding:**

British Heart Foundation, the UK Marfan Trust, the UK Marfan Association.

## Introduction

Marfan syndrome is a dominantly inherited disorder of connective tissue caused by mutations in the gene that encodes fibrillin-1.^1^ Cardiovascular complications, including aortic root dilatation, dissection, and rupture, are the leading cause of morbidity and mortality.^2^ β blockers have been advocated to reduce the rate of aortic root dilatation in people with Marfan syndrome.^3^, ^4^ Experimental models of Marfan syndrome suggest that angiotensin-II type 1 receptor blockers (ARBs) can alter biological pathways, including excessive TGF-β signalling, that might contribute to the pathogenesis of aortic complications,^5^, ^6^, ^7^, ^8^ a finding that is supported by observational data in clinical studies.^9^ Randomised trials in Marfan syndrome have compared the effects of the ARB losartan with either β blockers or control (where standard medical therapy could include β blockers) on aortic dilatation^10^, ^11^, ^12^, ^13^, ^14^, ^15^ without clear evidence of benefit. Other ARBs, such as irbesartan, might have greater bioavailability and a longer half-life than losartan with more potent antihypertensive effects. We aimed to determine the effects of the ARB irbesartan on the rate of aortic dilatation in children and adults with Marfan syndrome.

## Methods

### Study design and participants

The design and methods for the Aortic Irbesartan Marfan Study (AIMS) study have previously been reported.^16^ Briefly, AIMS was an investigator-led, placebo-controlled, double-blind randomised trial done at 22 centres with experience of managing Marfan Syndrome in the UK. The study protocol was approved by the UK National Research Ethics Committee, participating institutions, and relevant regulatory authorities. All participants, or their legal guardians in the case of children, provided written informed consent. The study complies with the principles of the Declaration of Helsinki.

Individuals were eligible for inclusion if they were aged between 6 and 40 years and had clinically confirmed Marfan syndrome using the revised Ghent diagnostic criteria^17^ and an aortic Z score of more than zero on baseline echocardiography. Individuals were excluded if they had undergone cardiac or aortic surgery or if this was planned, an aortic diameter of at least 4·5 cm, haemodynamically severe valve disease, a clear therapeutic indication or contraindication for ARB, or heart failure or they were pregnant. Individuals with potential for pregnancy could be enrolled if they were using a reliable means of contraception. Participants continued all their routinely indicated treatments. β-blocker use was not mandated by this protocol and was used at the discretion of the treating physician.

**Research in context**

**Evidence before this study**

The routine use of angiotensin receptor blockers to reduce aortic dilatation in Marfan syndrome has been controversial. Literature searches using PubMed (key words “Marfan syndrome“, “randomised“, and “angiotensin receptor blocker“) with no language or date restrictions (last search June 20, 2018) and discussion with other researchers were undertaken to identify all randomised controlled trials of angiotensin receptor blockers in Marfan syndrome. The largest randomised trial tested losartan against β blockers with similar effects on aortic dilatation, and the other two larger trials tested losartan against control with conflicting results. No trials of irbesartan in Marfan syndrome have been done.

**Added value of this study**

This AIMS trial shows that routine use of the angiotensin receptor blocker irbesartan was well tolerated and is associated with lower rates of aortic dilatation in children and young adults with Marfan syndrome compared with placebo. These results help to inform clinicians and patients about the use of irbesartan in Marfan syndrome.

**Implications of all the available evidence**

The results of the AIMS trial add to our knowledge of the effects of angiotensin receptor blockers in reducing the rate of aortic dilatation compared to placebo. Evidence suggests that this is a class effect and an individual patient data meta-analysis is in progress.

### Randomisation and masking

To ensure tolerability, all participants initially received open-label irbesartan 75 mg once daily for 4 weeks before randomisation. Participants were then randomly assigned, using a web-based system, 1:1 to irbesartan 150 mg once daily for 4 weeks, titrated up to 300 mg once daily if tolerated and weight was more than 50 kg, or matching placebo for up to 5 years. The randomisation sequence was generated with randomly varying block sizes of 2 or 4 and stratified by centre, participant's age and concurrent β-blocker use. Irbesartan and matching placebo were provided, in bulk, by Sanofi (Reading, UK) and drug packaging, storage and supply by Brecon Pharmaceuticals (Hay-on-Wye, UK).

### Procedures

At entry to the trial, before the open-label run-in phase, each participant had height, weight, blood pressure, heart rate, baseline electrocardiograph, current medication, and renal function studies recorded. If patients tolerated study medication and were willing to proceed with the study, full blood count and renal function were measured at baseline (at the time of randomisation), 1 month after entering the trial, and annually thereafter. In patients who provided consent, samples for fibrillin-1 mutation analysis were obtained if not taken already.

Transthoracic echocardiograms were acquired on an annual basis by experienced echocardiographers, according to a standardised research protocol and training provided by the core echocardiography laboratory, including assessment of inter-observer and intra-observer variability. Each echocardiogram was transferred in DICOM format to the core echocardiography laboratory at the John Radcliffe Hospital, University of Oxford, Oxford, where a single experienced investigator (XYJ) supervised the overall image analysis process and interpretation for the primary outcome data. The core echocardiography laboratory was blinded to study drug allocation to eliminate reading bias in echo measurement. Strict quality control processes were applied during the analysis according to the guidelines from the American Society of Echocardiography.^18^ From the parasternal long-axis view, aortic root diameter was measured using inner-edge to inner-edge technique during peak systole at the level of the sinus of Valsalva with the tip of the open cusps at ninety degrees to the direction of flow (primary endpoint; appendix p 9) and also at end diastole. Additional aortic diameter measurements were made and will be reported separately. To adjust for somatic growth, aortic Z score was calculated based on aortic sinus diameter and body surface area as previously described by Devereux and colleagues,^19^ and the Pettersen method^20^ was used as a sensitivity analysis.

### Outcomes

The primary outcome measure was the absolute change in aortic root diameter per year, measured by transthoracic echocardiography. Secondary outcomes reported here were annual rate of change in the Z score of aortic root diameter, occurrence of clinical events including aortic dissection, surgery for aortic dilatation death, and the incidence of adverse and serious adverse events.^16^ Additionally, effects of irbesartan on systolic and diastolic blood pressure over the follow-up period are reported. TGF-β samples were obtained from a subset of patients who provided consent at baseline and 1 year. Samples were analysed by use of ELISA and full methods are provided in the appendix. Serious adverse events were reported by investigators on specific forms and reviewed by adjudicators blinded to treatment allocation. Reasons for study withdrawals and treatment discontinuation and possible side-effects of treatment were also documented.

### Statistical analysis

On the basis of existing information on aortic root dilatation in Marfan syndrome, the original sample size was set up to detect a 0·5 mm reduction in the aortic root diameter on irbesartan compared with placebo with an SD of 1·8 mm. 490 participants were anticipated to detect this difference, assuming up to a 20% drop-out rate and 80% power. Recruitment was slower than expected and trial recruitment was terminated at 192 participants. The loss of power was mitigated by extending follow-up to a maximum of 5 years and statistical analyses for the rate of aortic dilatation that accounted for repeated measurements and missing outcome data.

The primary analysis was the comparison of the change in aortic root diameter per year between the irbesartan and placebo groups. The population in the intention-to-treat analysis for the primary and secondary outcomes included all randomly assigned patients according to their original treatment allocation. An additional analysis of the primary outcome was repeated including patients only up to the point that they were known to have stopped taking the trial treatment (per-protocol analysis). The mean annual rate of aortic root dilatation in each treatment group and the absolute difference in these rates was estimated using a linear mixed effects model for repeated measures.^21^ This model accounts for the baseline measure of aortic root diameter, while also incorporating all follow-up measurements, and enables the inclusion of participants with missing measurements. The model used in the primary analysis assumes that aortic root diameter changes linearly over time and a sensitivity analysis that relaxes this assumption was also done as follows: at each timepoint (from 1 to 5 years), the mean aortic root diameter in each treatment group, along with the absolute difference in these means, was estimated using a linear mixed effects model for repeated measures. This model accounts for the baseline measure of aortic root diameter.^22^ The model includes a continuous variable for time (in years), random intercepts and slopes, a linear interaction between time and treatment group, and assumes no effect of treatment at baseline. An unstructured variance-covariance matrix was used to allow for correlations between the random intercepts and slopes. The model was fitted using restricted maximum likelihood. Patients were followed for between 2 and 5 years and like all linear mixed models, the model assumes that when data on aortic root diameter were missing, they were missing-at-random. Further details of the model are provided in the appendix (p 3).

Similar models were used to examine the effect of irbesartan compared with placebo on the annual rates of change in aortic diastolic diameter, aortic Z score and systolic and diastolic blood pressure, and differences in TGF-β at 1 year. A small number of prespecified subgroup analyses were done for age, gender, blood pressure, β-blocker use, and aortic Z score by incorporating appropriate interaction terms. TGF-β was analysed in a subgroup of patients and was an exploratory post-hoc analysis. All statistical analyses were done on the intention-to-treat principle using Stata IC version 15.1.

This trial is registered with ISRCTN, number ISRCTN90011794.

### Role of the funding source

The funder of the study had no role in study design, data collection, data analysis, data interpretation, or writing of the report. The corresponding author had full access to all the data in the study and had final responsibility for the decision to submit for publication.

## Results

**Figure 1:**
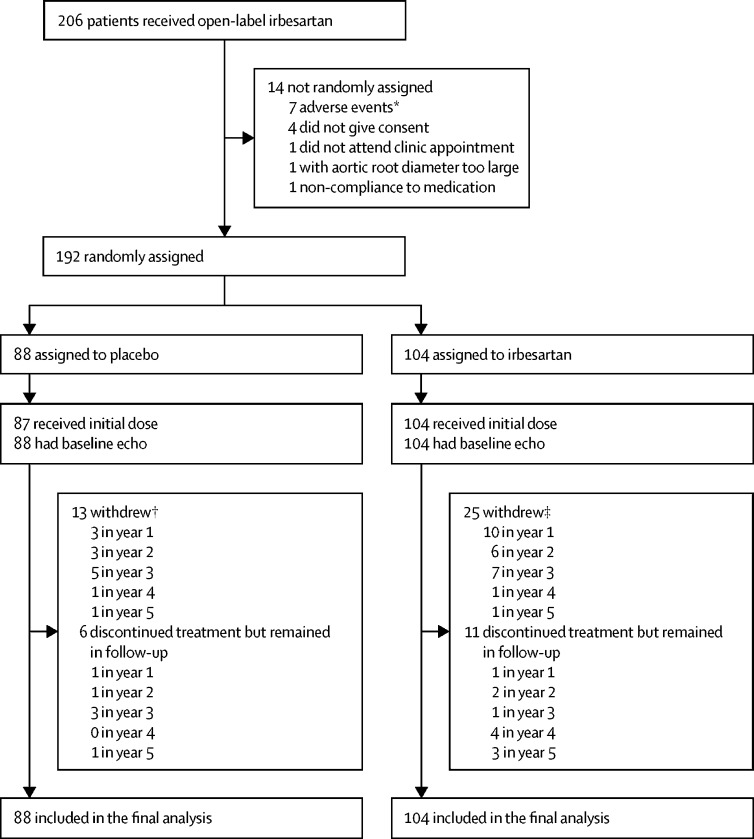
Study profile *These adverse events were not serious. †Four did not attend, four withdrew consent, three non-compliance to medication, and two investigator decisions. ‡Nine did not attend, five withdrew consent, five non-compliance to medication, three investigator decisions, and three were ineligible.

Between March 14, 2012, and May 1, 2015, 192 participants were recruited; 104 were randomly assigned to irbesartan and 88 to placebo (figure 1). Participants were followed for a median of 4 years (IQR 3–5), with the final patient visit on March 12, 2018. Baseline characteristics were well balanced between the groups (table 1); participants had a median age of 18 years (12–28), 99 (52%) were female, mean blood pressure was 110/65 mm Hg (SDs 16 and 12), and 108 (56%) participants were taking β-blocker treatment at baseline. Of the 149 patients who agreed to fibrillin-1 gene mutation analysis, 138 (93%) were confirmed as having positive mutations. More withdrawals occurred in the irbesartan group than in the placebo group, although they did not appear to be related to any side-effects of treatment. Among the participants remaining in follow-up, discontinuation of study medication did not differ between the two groups (appendix p 5). A dose of 300 mg of irbesartan was achieved in 80% of participants with no apparent difference related to age or weight (appendix pp 5–6).

**Table 1:** Baseline characteristics

		**Placebo, n=88**	**Irbesartan, n=104**
Age (years)			
	6 to <11	19 (22%)	21 (20%)
	11 to <18	26 (30%)	27 (26%)
	18 to <25	13 (15%)	22 (21%)
	25 to <41	30 (34%)	34 (33%)
Sex			
	Female	42 (48%)	57 (55%)
	Male	46 (52%)	47 (45%)
Ethnicity			
	White British, Irish, or other	78 (89%)	97 (93%)
	Asian	6 (7%)	2 (2%)
	Black African or Caribbean	2 (2%)	1 (1%)
	Mixed	1 (1%)	2 (2%)
	Other	1 (1%)	2 (2%)
Aortic root diameter, mm		34·4 (5·8)	34·4 (5·5)
Aortic Z score		3·3 (2·1)	3·2 (2·0)
β-blocker use		52 (59%)	56 (54%)
Systolic blood pressure, mm Hg		109 (15)*	110 (16)
Diastolic blood pressure, mm Hg		64 (11)*	66 (12)
Heart rate		72 (13)*	72 (14)
Height, cm		178 (161–186)	175 (163–187)
Weight, kg		60·7 (44·6–73·1)	61·6 (44·0–76·8)
Body-mass index, kg/m^2^		18·9 (16·0–22·4)	19·3 (15·8–22·5)
Body surface area, m^2^		1·78 (1·50–1·96)	1·78 (1·44–2·01)
Lower segment, cm		94 (84–100)*	94 (86–100)
Arm span, cm		181 (164–190)†	179 (163–192)
Creatinine, μmol/L		56 (14)*	56 (16)*

Data are n (%), mean (SD), or median (IQR).

* One patient with missing data.

† Two patients with missing data.

**Figure 2:**
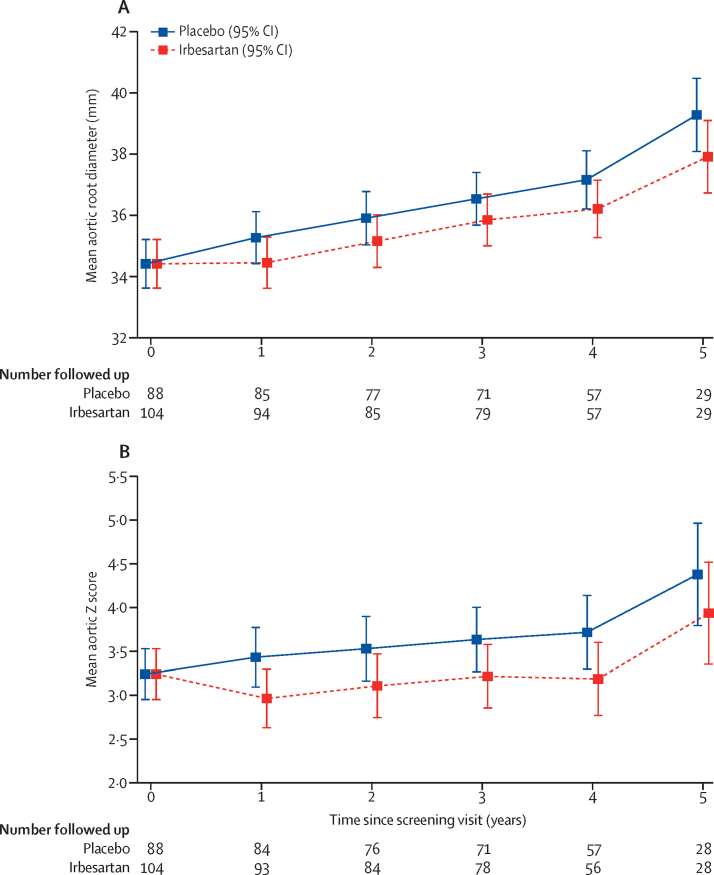
Aortic diameter measurements over time (A) Mean aortic root diameter over time. (B) Mean aortic Z score over time. Means were estimated by use of a linear mixed effects model for repeated measures. At the final follow up visit the majority of patients had not reached 5 years of study participation, which accounts for the sharp decrease in numbers available for follow-up between year 4 and 5.

At baseline, mean aortic root diameter was 34·4 mm (SD 5·6; table 2). The annual rate of change in aortic root systolic diameter was lower in the irbesartan group than in the placebo group (0·53 mm per year [95% CI 0·39 to 0·67] *vs* 0·74 mm per year [0·60 to 0·89]; difference in means −0·22 mm per year [–0·41 to −0·02], p=0·030). At 3 years the estimated difference in the mean aortic root diameters was −0·67 mm (95% CI −1·32 to −0·03, p=0·041; figure 2A; table 2). A similar finding was also apparent when diastolic diameters were used (0·62 mm per year [95% CI 0·49 to 0·76] in the irbesartan group *vs* 0·82 mm per year [0·68 to 0·96] in the placebo group; difference in means −0·20 mm per year [–0·39 to −0·01], p=0·038; appendix p 6). The per-protocol analysis based on measurements to the point that treatment was discontinued showed similar results to the intention-to-treat analysis (difference in means −0·21 mm per year, 95% CI −0·40 to −0·02, p=0·027).

**Table 2:** Aortic root diameter

		**Placebo**	**Irbesartan**	**Difference (95% CI)**	**p value**
Change in aortic root systolic diameter, mm per year, mean (95% CI)		0·74 (0·60 to 0·89)	0·53 (0·39 to 0·67)	−0·22 (−0·41 to −0·02)	0·030
Aortic root diameter, mm					
	Baseline, mean (SD), n	34·4 (5·8), 88	34·4 (5·5), 104	..	..
	1 year, mean (95% CI), n	35·3 (34·4 to 36·1), 85	34·5 (33·6 to 35·3), 94	−0·82 (−1·33 to −0·30)	0·0017
	2 years, mean (95% CI), n	35·9 (35·0 to 36·8), 77	35·2 (34·3 to 36·0), 85	−0·75 (−1·41 to −0·10)	0·024
	3 years, mean (95% CI), n	36·5 (35·7 to 37·4), 71	35·9 (35·0 to 36·7), 79	−0·67 (−1·32 to −0·03)	0·041
	4 years, mean (95% CI), n	37·1 (36·2 to 38·1), 57	36·2 (35·3 to 37·1), 57	−0·95 (−1·86 to −0·05)	0·038
	5 years, mean (95% CI), n	39·2 (38·0 to 40·4), 29	37·8 (36·7 to 39·0), 29	−1·37 (−2·83 to 0·08)	0·065

Comparisons are made between groups at each timepoint. At the final follow-up visit the majority of patients had not reached 5 years of study participation, which accounts for the sharp decrease in numbers available for follow-up between year 4 and 5.

The annual rate of change of aortic root Z scores was also lower in the irbesartan group (0·05 per year [95% CI −0·02 to 0·11] *vs* 0·15 per year [0·08 to 0·22]; difference in means −0·10 per year [–0·19 to −0·01], p=0·035; table 3). Results for mean rate of change in Z score for irbesartan compared with placebo based on the Pettersen formula^20^ were very similar (−0·10 per year, 95% CI −0·18 to −0·01, p=0·028). At 3 years, the estimated difference in the mean aortic Z scores was −0·41 mm (95% CI −0·73 to −0·08, p=0·013; figure 2B; table 3).

**Table 3:** Aortic Z score

		**Placebo**	**Irbesartan**	**Difference (95% CI)**	**p value**
Change in aortic Z score (per year), mean (95% CI)		0·15 (0·08 to 0·22)	0·05 (−0·02 to 0·11)	−0·10 (−0·19 to −0·01)	0·035
Aortic Z score					
	Baseline, mean (SD); n	3·28 (2·10); 88	3·21 (2·00); 104	..	..
	1 year, mean (95% CI), n	3·44 (3·10 to 3·77), 84	2·96 (2·63 to 3·30), 93	−0·47 (−0·74 to −0·20)	0·0006
	2 years, mean (95% CI), n	3·53 (3·16 to 3·90), 76	3·11 (2·75 to 3·47), 84	−0·42 (−0·74 to −0·10)	0·010
	3 years, mean (95% CI), n	3·63 (3·26 to 4·00), 71	3·22 (2·86 to 3·59), 78	−0·41 (−0·73 to −0·08)	0·013
	4 years, mean (95% CI), n	3·72 (3·30 to 4·14), 57	3·18 (2·76 to 3·60), 56	−0·54 (−0·98 to −0·10)	0·016
	5 years, mean (95% CI), n	4·38 (3·80 to 4·97), 28	3·94 (3·35 to 4·52), 28	−0·45 (−1·14 to 0·25)	0·21

Comparisons are made between groups at each timepoint. At the final follow-up visit the majority of patients had not reached 5 years of study participation, which accounts for the sharp decrease in numbers available for follow-up between year 4 and 5.

**Figure 3:**
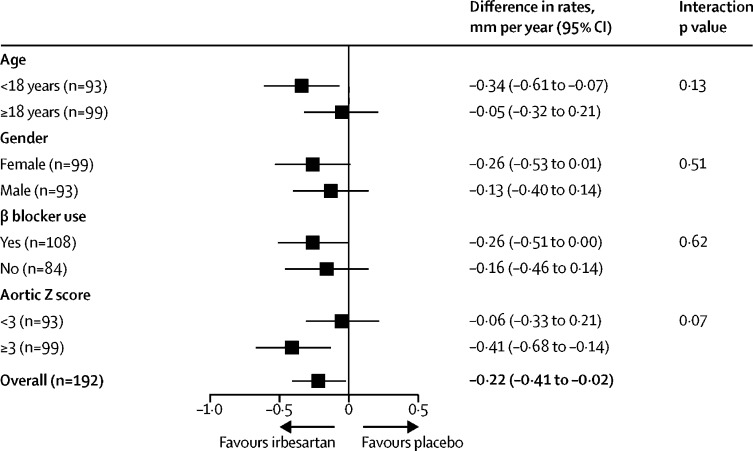
Differences in rate of aortic root diameter change

There was no evidence for an interaction in any of the prespecified subgroup analyses. Specifically, there was no evidence of interaction with concurrent β blocker use. Irbesartan could have greater effects in younger participants and those with a higher aortic Z score at baseline, but no statistical evidence supported this (figure 3). TGF-β concentrations from baseline to 1 year for 99 patients did not differ between groups (appendix p 7).

In the placebo group, both systolic and diastolic blood pressure consistently increased during the study, whereas blood pressure was reduced in the irbesartan group. The difference at 1 year (irbesartan compared with placebo) was −6·3 mm Hg (95% CI −9·8 to −2·9) in systolic blood pressure and −3·6 mm Hg (−6·5 to −0·8) in diastolic blood pressure which was maintained throughout the trial (appendix pp 8, 10).

The numbers of patients reporting at least one serious adverse event were 21 (24%) in the irbesartan group and 24 (23%) in the placebo group. No differences in the occurrence of any serious adverse events, or those classified as cardiovascular, was observed between irbesartan and placebo (appendix pp 8–9). There were five aortic surgical procedures in the irbesartan group (two elective aortic root repairs and three elective external stent procedures) and four in the placebo group (three elective aortic root repairs and one emergency aortic dissection). There were no deaths during the trial.

## Discussion

This study provides evidence of a reduction in the rate of aortic root dilatation in children and young adults with Marfan syndrome treated with irbesartan compared with placebo, over a 5-year observation period. The treatment was well tolerated, and no differences in adverse events due to suspected side-effects were observed between irbesartan and placebo groups.

The effects of irbesartan on reduction of aortic dilatation appear to occur early and to be maintained over time compared with placebo These effects are mirrored by effects on blood pressure, suggesting a possible association. The apparent increase in aortic diameter after year 4 in both groups might be an artifact due to the reduced numbers available for follow-up during that period, although the observed reduction in aortic root diameter in the irbesartan group remained.

Our study had a robust double-blind design with use of a placebo control, an independent core laboratory blinded to treatment to evaluate echocardiographic endpoints, high proportions of children, more than 50% treatment with β blockers and good compliance to trial treatment. We provide evidence of a reduction in the rate of aortic dilatation in participants receiving irbesartan that was apparent at 1 year and maintained for the duration of the study. In our cohort of both children and adults, we used the rate of change in the absolute diameter as the primary endpoint, because it was a more direct measure. Our findings were similar for measurements of diastolic aortic root diameter and Z score, which take somatic growth over time into account. There has been discussion about the most appropriate method to estimate Z score in the Marfan population.^23^ These methods for estimating aortic Z score have been primarily developed and validated in a population that shows somatic growth and might not be reliable in adults. We used the Devereux method for the primary Z score analysis and the Pettersen method for sensitivity analysis, which yielded very similar results for differences in rate of change between irbesartan and placebo.

Previous randomised studies of ARBs in Marfan syndrome have all assessed the impact of losartan against either β blocker, placebo-control or open control.^10^, ^11^, ^12^, ^13^, ^14^, ^15^ Groenink and colleagues^13^ reported a beneficial effect of open label losartan over 3 years of about 0·2 mm per year which is similar to our finding. The study only included adults with established aortic root dilatation, in contrast to our study, in which half of all participants were younger than 18 years. Milleron and colleagues^12^ showed a modest, but not statistically significant, reduction in aortic dilatation with losartan compared with placebo in 303 patients with Marfan syndrome followed for 3·5 years.

β blockers might not be tolerated in some Marfan patients with asthmatic symptoms and might paradoxically worsen vascular stiffness.^24^ β blockers and ARBs are therefore not competitive and if effective, ARBs might be synergistic or additive to standard β-blocker therapy. In the US Pediatric Network Trial of losartan versus atenolol among 608 participants with Marfan syndrome aged 6 months to 25 years,^10^ the rate of aortic dilatation did not differ between the two groups. The Pediatric Heart Network Trial is the largest randomised controlled trial in Marfan syndrome and suggests that ARBs and β blockers might have similar effects on aortic dilatation.^10^ In the AIMS trial, β blockers were provided according to clinical need and about half the patients in each group were on β blockers at baseline. We used a placebo control rather than a β blocker, although β blockers were used according to clinical indication in more than half the patients. We found no evidence of interaction of irbesartan effect with β blockers or in any prespecified subgroup, although the study was underpowered in this regard.

Unlike other studies, AIMS assessed the effects of irbesartan in Marfan syndrome. Irbesartan is a selective angiotensin type-1 receptor blocker with greater bioavailability and a longer half-life than losartan (11–15 h for irbesartan *vs* 6–9 h for losartan), with more powerful antihypertensive effects.^25^, ^26^ Irbesartan might also have effects on the pathophysiology of aortic disease, including TGF-β pathways.^7^ Another possibility is that effects on aortic dilatation reflect reduced blood pressure. Milleron and colleagues^12^ showed that losartan resulted in a similar reduction in systolic blood pressure compared with the control, as we observed in the AIMS trial, without a clear effect on the rate of aortic dilatation. This suggests other pathways might be involved in the clinical effect, although patients were also on average of 10 years older than those in this study. We also found a reduced rate of change of diastolic aortic diameter which is likely to be less dependent on ambient blood pressure. Subgroup analyses showed numerically greater reductions with irbesartan in younger participants and those with established aortic dilatation at baseline, but they were not statistically significant and will require confirmation in other studies. About 40% of patients contributed to TGF-β analysis, and the groups did not differ from baseline to 1 year. Effects on reducing aortic dilatation in Marfan syndrome might be a class effect among ARBs, and any differences observed across the trials might be related to patient selection, doses achieved, duration of treatment, and precision of measurement method, which will be further assessed in a planned individual patient data meta-analysis.^27^

AIMS was neither designed nor powered to detect differences in clinical outcomes between the groups and the primary outcome of aortic diameter is a surrogate measure. In clinical practice, aortic diameter measured by echocardiography is usually used to determine the point at which surgical intervention is indicated, and we postulate that reduced rate of aortic dilatation with irbesartan might delay the need for surgery and improve outcomes, although the clinical effect of these modest differences remains unclear. Additionally, irbesartan was safe and well tolerated in both children and adults with no difference in the number of adverse events or withdrawals due to side effects between the two groups.

This study had some limitations. The trial was unable to enrol the original sample size of 490 participants. Extending follow-up and statistical analysis that used the correlations between baseline and follow-up measures improved the precision of the estimated treatment effects and helped to preserve power, although the smaller than planned sample size remains a limitation. This resulted in a SD of 0·53 mm per year for the rate of aortic dilatation, compared with the 1·8 mm used to compare follow-up aortic root diameters in the original sample size estimation. By chance more participants were assigned to the irbesartan group due to stratification by centre, age, and β blocker use, the lower than planned sample size, and incomplete randomisation blocks because several centres enrolled only a few participants.

In summary, in this double-blind, placebo-controlled randomised controlled trial, we have shown that irbesartan can slow the rate of aortic dilatation in children and adults with Marfan syndrome and is well tolerated. The clinical implications of this observation need further investigation, but our data provide evidence to support the use of irbesartan to reduce the rate of aortic dilatation in this relatively common and potentially fatal inherited condition.

## Data sharing

Data from the AIMS trial, including patient-level data, can be made available on request to established research groups for the purpose of improving human health with an appropriate data sharing agreement. Please contact the corresponding author for data sharing.
