# Supplementary material for: Irbesartan in Marfan syndrome (AIMS): a double-blind, placebo-controlled randomised trial
Source: Lancet. 2019 Dec 21;394(10216):2263–70. doi: 10.1016/S0140-6736(19)32518-8 (PMC6934233; doi:10.1016/S0140-6736(19)32518-8)
Supplement: Supplementary appendix [file mmc1.pdf]

THE LANCET

Supplementary appendix

This appendix formed part of the original submission and has been peer reviewed. We post it as supplied by the authors.

Supplement to: Mullen M, Jin XY, Child A, et al. Irbesartan in Marfan syndrome (AIMS): a double-blind, placebo-controlled randomised trial. *Lancet* 2019; published online Dec 10. http://dx.doi.org/10.1016/S0140-6736(19)32518-8.

SUPPLEMENTARY APPENDIX

Effects of Irbesartan on Aortic Root Dilatation in Marfan syndrome

The Aortic Irbesartan Marfan Study (AIMS)

Collaborating centres and staff (Principal Investigators in bold)

Aberdeen Royal Infirmary (5)
John Dean, Bartosz Was, Heather Gow, Jane Murray, Mariella D'Allessandro, Michael Christie, Patricia Cooper, Philip Booth, Sharon Burns, Yvonne Paterson

Birmingham Children's Hospital (2)
Ashish Chikermane, Anthony Assing, Catherine Cotter, Gillian Atkins, Helen Williamson

Cumberland Infirmary (1)
Justin Barclay, Alan Jennison, Alex Henderson, Anna McSkeane, Helen Fairlamb, Julie Kelly, Nicola Kelsall, Scott Prentice

Freeman Hospital (5)
John O'Sullivan, Alison Head-Baister, Angela Phillipson, Anna Johnson, D Crossland, Jack Oliver, Jade Davison, Jill Wake, Louise Quinn, Maureen Foreman, Vera Wealleans

Golden Jubilee National Hospital (1)
Niki Walker, Alexis Duncan, Evelyn Tibbs, Ruth Kelly

Great Ormond Street Hospital (6)
Sachin Khambadkone, Bridget Zotti, Cassie Brady, Elena Cervi, Ella Field, Eszter Szepezvary, Florence Mantey, Gillian Riley, Heather Titmus, Ilaria Bo, Juan Pablo Kaski, Loren Green, Nigel Jones, Rebecca Banks

Guy's and St Thomas' (13)
Christopher Kiesewetter, **Sujeev Mathur**, Alessandra Frigiola, Alex Savis, Holly Belfield, Josephine Guzman, Julia Harris, Karen Wilson, Kelly Peacock, Kirsty Gibson, Paul Wellman, Professor John Simpson , Saleha Kabir, Sitali Mushemi

James Cook University Hospital (12)
Michael Stewart, Bev Atkinson, Cath Richardson, Elaine Leng, Paul Brennan

Leeds General Infirmary (20)
John Thomson, Annabel Nixon, Collette Spencer, James Oliver, Jan Forster, Louise Turner, Samantha Bainbridge

Ninewells Hospital and Medical School (2)
Anna Maria Choy, Adelle Dawson, Gwen Kiddie, Heather Kerr, Ify Mordi, Jackie Duff, Jacqueline Dunlop, Jonathan Berg, Pauline Armory

Norfolk and Norwich University Hospitals (4)
Leisa Freeman, Amir Anwar, Charles Graham, Clare London, Gail Healey, Ian Gallagher, Mary Isley, Rizwan Ahmed, Sheila Wood

Northern General Hospital (2)
Nigel Wheeldon, Cecilia Mason, Farook Nassim, Janet Middle, Justin Adams, Karen Angelini, Kay Housley, Kim Ryalls, Michael Agyemang, Rachel Walker, Robina Batigan, Tina Bennett

Queen Elizabeth Hospital Birmingham (11)
Paul Clift, Amor Mia B. Alvior, Annette Nilsson, Carole Green, Charlotte Crook, Connie Becani Palmer, Elizabeth Dwenger, Phillipa Doherty, Rebecca Igbokwe, Saba Sharif, Sonia MacDonald

Royal Brompton Hospital (17)
Lorna Swan, Cathy West, Kevin Kirby, Nitha Naqvi, Sophie Welch, Suad Warsama, Wei Li, Zohreh Farzad

Royal Hospital for Children, Glasgow (10)
Ben Smith, **Victoria Murday**, Alexis Duncan, Eamonn Murtagh, Emma Adams, Lesley Armour, Stuart Lilley

St Bartholomew's Hospital (11)
Bejal Pandya, Amy Richards, Mervyn Andiapen, Rebecca Macrae

St George's Hospital (26)
Anne Child, Maite Tome, Carmel Hutchinson, Kameka Angulo, Rooba Kauppayamootoo, Sabiha Gati, Elizabeth Cruddas

St Mary's Hospital, Manchester (2)
William G Newman, Catherine Breen

University Hospital of Wales (4)
Dhavendra Kumar, **Dirk G Wilson**, Adele Farrugia, Alan Fraser, Jayne Sumers, Jessie Powell, Julie Edwards, Terese Hale, Zoe Boult

University Hospital Southampton (6)
Aisling Carroll, **Gruschen Veldtman**, Andrew Ho, David Black, Lisa Fletcher, Sue Mapstone, Tara Bharucha

University Hospitals Bristol (30)
Graham Stuart, Gary Marsh, Joanne Jones, Karen Sheehan, Kathleen Selway, Kirsty Stevenson, Martin Nelson, Rebecca Fairweather, Stephanie Curtis, Sue Simpson

Western General Hospital (2)
Martin Denvir, Audrey White, Jill Steven, Joanna Munro, Wayne Lam

Trial Steering Committee

David Crossman (Independent Chair), Anne Child, Tim Clayton, Marcus Flather, Ira Jakupovic (Sponsor representative), Xu Yu Jin, Rosemary Knight, Michael Mullen, Graham Stuart, Laura Van Dyck, Shannan Amoils (British Heart Foundation)

Data Monitoring Committee

William Toff (Chair), Mario Petrou, Paul Silcocks, Raymond MacAllister. Statistical reports provided by Matthew Dodd and Thomas Godec

Clinical Event Adjudicators

Michael Mullen, Graham Stuart

Additional details for statistical analysis

The formula used for the statistical model for the primary analysis is as follows:

$$Y_{ij} = (\beta_0 + u_{0j}) + (\beta_1 + \beta_2 treatment_j + u_{1j}) time_i + e_{ij}$$

Where:

- $i = 0, \dots, m$, is the ith time-point (in years) out of m time-points
- $j = 1, \dots, n$, is the jth participant out of a total of n participants in the trial
- Y_{ij} is the aortic root diameter of the jth participant at the ith time-point (in years)
- $treatment_j$ is 0 if the jth participant is in the placebo group and 1 if they are in the active (Irbesartan) group
- β_0 is the estimated mean aortic root diameter at baseline
- β_1 is the estimated mean annual rate of change in aortic root diameter in the placebo group
- β_2 is the estimated difference in the mean annual rates between the active (Irbesartan) and placebo groups
- $\begin{pmatrix} u_{0j} \\ u_{1j} \end{pmatrix} \sim N\left(\begin{pmatrix} 0 \\ 0 \end{pmatrix}, \Sigma_u\right)$ are the random effects and $\Sigma_u = \begin{pmatrix} \sigma_{u_{00}}^2 & \sigma_{u_{01}} \\ \sigma_{u_{01}} & \sigma_{u_{11}}^2 \end{pmatrix}$
- $e_{ij} \sim N(0, \sigma_e^2)$ are the error terms

Methods for TGF-beta measurement

Whole blood was collected from an antecubital forearm vein into a serum separator tube (SST) and allowed to clot for 30 minutes at room temperature. For complete release of TGF-1, the samples were refrigerated overnight at 2-8°C. Following overnight incubation, samples are centrifuge for 15 minutes at 1,000g. The resulting serum was decanted and stored frozen at -70°C. Stored samples were thawed at room temperature. Samples required activation by addition of 20 mL of 1N HCl, mixed by vortexing and allowed to incubate at room temperature for 10 minutes. Acidified samples are then neutralised by addition of 20 mL 1.2 N NaOH/0.5 M HEPES. Prior to assaying, activated samples were diluted 20 fold in the manufactures calibrator diluent. The TFG-b was determined by enzyme linked immunosorbent assay (R&D Systems, Abingdon, Oxfordshire, UK). 50 mL of assay diluent is added to each well of the supplied 96-well microtitre plate. 50 mL of standard, control, or activated sample was added to the well. The plate was incubated for 2 hours at room temperature on an automated plate shaker. Following incubation each well was aspirated and washed, repeating the process three times for a total of four washes using 400 mL of wash buffer. After the last wash any remaining wash buffer is removed by decanting and inverting the plate and blotting against clean paper towels. 100 mL of TGF-b conjugate is added to each well. The plate was further incubated for 2 hours at room temperature. The aspiration and wash was repeated a further 4 times. 100 mL of substrate solution was then added to each well and incubated for 30 minutes at room temperature in the dark. 100 mL of stop solution was added to each well, tapping the plate to ensure thorough mixing. The optical density was read within 30 minutes, using a microplate reader set to 450 nm with wavelength correction set to 540 nm or 570 nm. The TG3F-b assay has a reportable range of 1.7 to 2,000 ng/L. Total precision was 6.4 to 9.3% in the range 79 to 730 ng/L.

Table S1: Tolerability of trial medication over time

	Year 1		Year 2		Year 3	
	Placebo	Irbesartan	Placebo	Irbesartan	Placebo	Irbesartan
Number attending visit	85	93	80	88	75	80
Number taking medication	83	88	76	81	70	76
Number tolerating medication	82	86	75	81	68	75

	Year 4		Year 5	
	Placebo	Irbesartan	Placebo	Irbesartan
Number attending visit	62	60	31	29
Number taking medication	56	53	27	23
Number tolerating medication	56	52	27	23

There were no statistical differences between groups at any time point
At the final follow up visit the majority of patients had not reached five years of study participation which accounts for the sharp decrease in numbers available for follow up between year 4 and 5

Table S2: Peak dosage tolerated by body weight

	Peak dosage tolerated (mg)			
**Weight at baseline**	**75**	**150**	**300**	**Total**
**<50 kg**				
Placebo	0	3	23	26
Irbesartan	0	5	25	30
Overall	0	8	48	56
**≥50 kg**				
Placebo	2	10	49	61
Irbesartan	5	13	56	74
Overall	7	23	105	135
**Total**	7	31	153	191

Weight values provided are at study entry. Patients generally gained weight during the course of the study and if they transitioned from <50kg to >50kg this allowed up- titration to 300mg if tolerated.

Table S3: Peak dosage tolerated by age

	Peak dosage tolerated (mg)			
**Age at baseline**	**75**	**150**	**300**	**Total**
**<18 years**				
Placebo	1	6	38	45
Irbesartan	0	10	38	48
Overall	1	16	76	93
**≥18 years**				
Placebo	1	7	34	42
Irbesartan	5	8	43	56
Overall	6	15	77	98
**Total**	7	31	153	191

Table S4. End diastolic aortic root diameter in the placebo and irbesartan groups over time

	**Rate of change in end diastolic aortic root diameter (mm/year), mean (95% CI)**		**Difference in rates (mm/year) (95% CI)**
	Placebo	Irbesartan	
	0.82 (0.68 to 0.96)	0.62 (0.49 to 0.76)	-0.20 (-0.39 to -0.01); p = 0.038
	**End diastolic aortic root diameter (mm), mean (95% CI)**		**Difference in means (95% CI)**
	Placebo	Irbesartan	
**Baseline, mean (SD)**<br>**N**	32.9 (5.9)<br>88	33.1 (5.6)<br>104	
**1 year**<br>**N**	33.9 (33.1 to 34.8)<br>85	33.1 (32.3 to 34.0)<br>94	-0.81 (-1.35 to -0.28); p = 0.003
**2 years**<br>**N**	34.6 (33.7 to 35.5)<br>77	34.0 (33.2 to 34.9)<br>85	-0.59 (-1.23 to 0.06); p = 0.076
**3 years**<br>**N**	35.4 (34.5 to 36.2)<br>71	34.8 (33.9 to 35.6)<br>79	-0.60 (-1.24 to 0.03); p = 0.063
**4 years**<br>**N**	36.2 (35.3 to 37.2)<br>57	35.2 (34.3 to 36.2)<br>56	-1.00 (-1.91 to -0.09); p = 0.030
**5 years**<br>**N**	37.6 (36.5 to 38.8)<br>29	36.6 (35.4 to 37.8)<br>29	-1.02 (-2.45 to 0.42); p = 0.16

CI = confidence interval; SD= standard deviation
Comparisons are made between groups at each time point.

Table S5: TGF beta values at baseline and one year

	Log TGF-beta (log(ng/L)), mean (95% CI)		Difference in means, mean (95% CI)
	Placebo	Irbesartan	
**Baseline**	5.43 (5.29 to 5.58)<br>N=30	5.43 (5.29 to 5.58)<br>N=37	-
**1 Year**	5.40 (5.24 to 5.55)<br>N=42	5.29 (5.14 to 5.45)<br>N=47	-0.10 (-0.25 to 0.05)<br>p = 0.18

Table S6. Systolic and diastolic blood pressure in the placebo and irbesartan groups over time

	Systolic blood pressure (mmHg), mean (95% CI)		Difference in means (95% CI), p
	Placebo	Irbesartan	
**Baseline, mean (SD) N**	109.0 (15.3) 87	110.4 (15.8) 104	
**1 year N**	115.3 (112.5 to 118.2) 84	109.0 (106.2 to 111.7) 93	-6.3 (-9.8 to -2.9); p<0.001
**2 years N**	116.2 (113.3 to 119.1) 79	110.1 (107.4 to 112.9) 88	-6.1 (-9.6 to -2.5); p = 0.001
**3 years N**	116.4 (113.7 to 119.2) 74	110.5 (107.8 to 113.1) 78	-6.0 (-9.4 to -2.6); p = 0.001
**4 years N**	117.8 (114.8 to 120.7) 60	110.9 (107.9 to 113.9) 58	-6.8 (-10.8 to -2.8); p = 0.001
**5 years N**	119.8 (115.8 to 123.8) 28	110.1 (106.1 to 114.1) 28	-9.7 (-14.9 to -4.5); p<0.001
	**Diastolic blood pressure (mmHg), mean (95% CI)**		**Difference in means (95% CI)**
	Placebo	Irbesartan	
**Baseline, mean (SD) N**	64.0 (11.0) 87	65.6 (12.1) 104	
**1 year N**	70.0 (67.8 to 72.3) 85	66.4 (64.2 to 68.5) 93	-3.6 (-6.5 to -0.8); p = 0.013
**2 years N**	70.8 (68.7 to 72.9) 79	67.1 (65.1 to 69.1) 88	-3.7 (-6.4 to -1.0); p = 0.007
**3 years N**	71.2 (69.1 to 73.3) 74	65.6 (63.6 to 67.6) 78	-5.6 (-8.3 to -2.9); p<0.001
**4 years N**	72.1 (70.0 to 74.2) 60	66.5 (64.4 to 68.7) 58	-5.5 (-8.4 to -2.7); p<0.001
**5 years N**	73.7 (70.9 to 76.5) 28	66.2 (63.4 to 69.0) 28	-7.5 (-11.3 to -3.7); p<0.001

CI = confidence interval; SD= standard deviation
Comparisons are made between groups at each time point.

Table S7. Serious Adverse Events (SAEs)

	**Placebo (n = 88)**	**Irbesartan (n = 104)**
Number with ≥1 SAE	21 (23.9%)	24 (23.1%)
Number with no SAE	67 (76.1%)	80 (76.9%)

Table S8. Cardiac and non-cardiac Serious Adverse Events (SAEs)

	Placebo (n = 88)	Irbesartan (n = 104)
Cardiac SAEs<br>N	8 (9.1%);<br>9	8 (7.7%);<br>10
Non-cardiac SAEs<br>N	16 (18.2%);<br>32	19 (18.3%);<br>27
Total<br>N	21 (23.9%);<br>41	24 (23.1%);<br>37

Data are presented as the number (%) of participants with an SAE; total number of SAEs. Of the cardiac SAEs there were 5 aortic surgical procedures in the irbesartan group (2 elective aortic root repairs and 3 elective external stent procedures) and 4 in the placebo group (3 elective aortic root repairs and one emergency aortic dissection) and there were no deaths during the trial.

Figure S1. Measurement of aortic diameter: the yellow arrow line indicates the maximal diameters at the aortic sinus level.

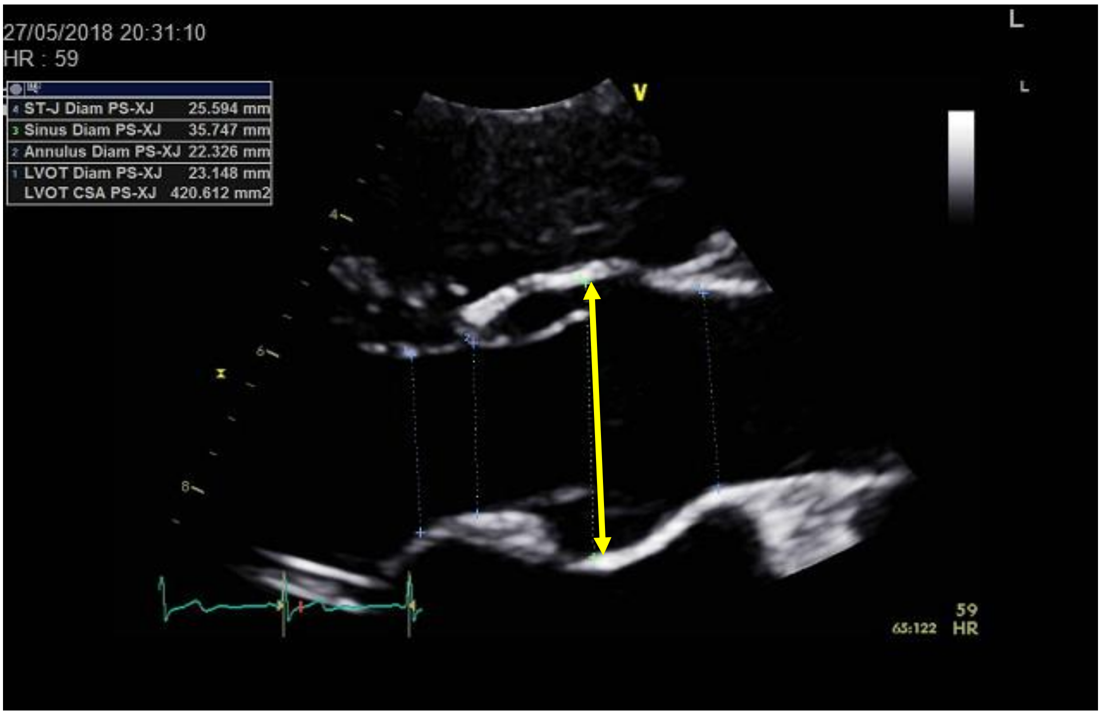

Figure S2. Panel A. Mean systolic blood pressure over time

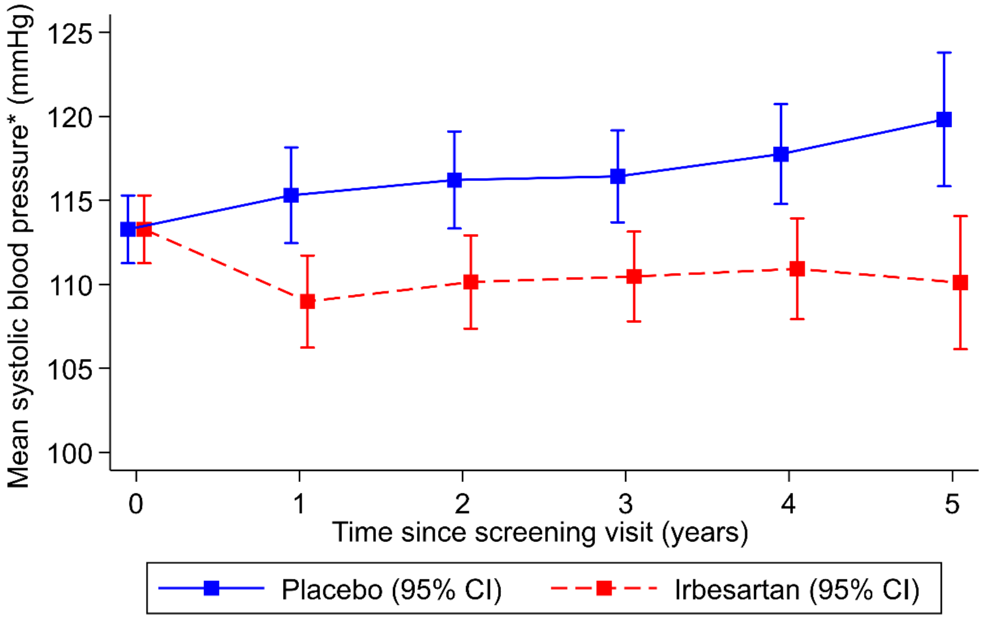

Number followed up

Placebo	87	84	79	74	60	28
Irbesartan	104	93	88	78	58	28

Panel B. Mean diastolic blood pressure over time

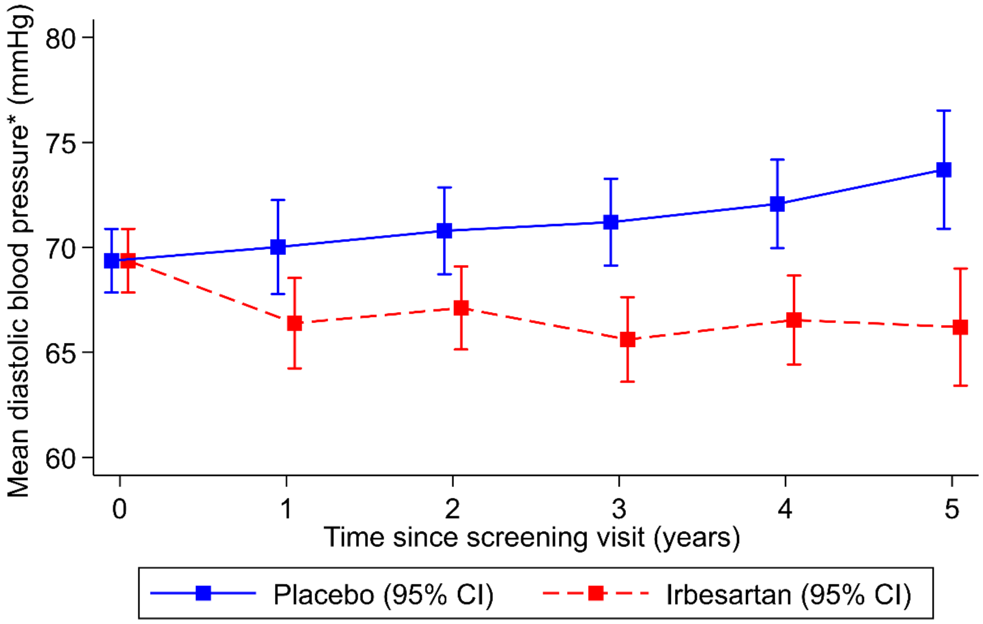

Number followed up

Placebo	87	85	79	74	60	28
Irbesartan	104	93	88	78	58	28

* estimated using a linear mixed effects model for repeated measures. CI – confidence interval.

Figure S3. Mean end diastolic aortic root diameter over time

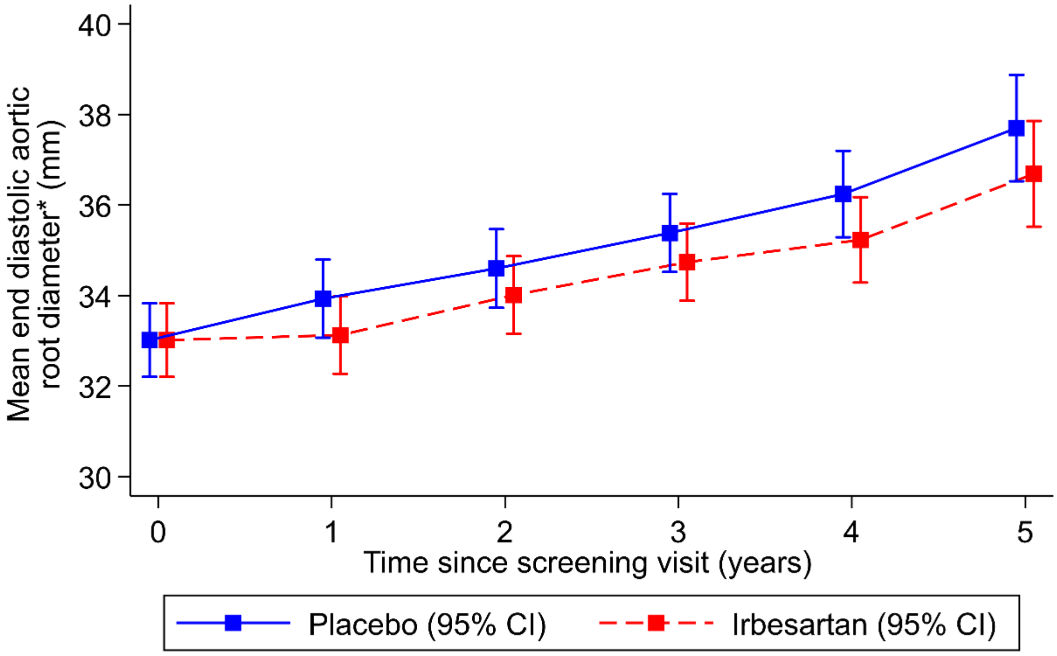

Number followed up						
Placebo	88	85	77	71	57	29
Irbesartan	104	94	85	79	56	29

* estimated using a linear mixed effects model for repeated measures. CI – confidence interval.
